# Formation of Calprotectin Inhibits Amyloid Aggregation of S100A8 and S100A9 Proteins

**DOI:** 10.1021/acschemneuro.4c00093

**Published:** 2024-04-18

**Authors:** Ieva Baronaitė, Darius Šulskis, Aurimas Kopu̅stas, Marijonas Tutkus, Vytautas Smirnovas

**Affiliations:** † Institute of Biotechnology, Life Sciences Center, Vilnius University, LT-10257 Vilnius, Lithuania; ‡ Department of Molecular Compound Physics, Center for Physical Sciences and Technology, LT- 10257 Vilnius, Lithuania

**Keywords:** aggregation, amyloid, S100, inflammation

## Abstract

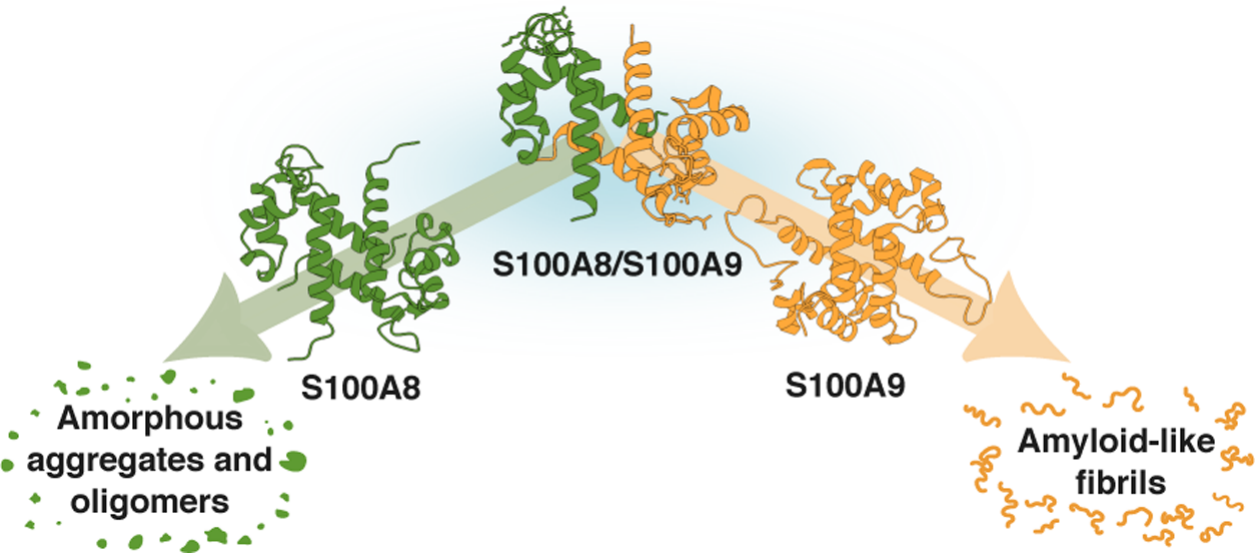

Calcium-binding S100A8 and S100A9 proteins play a significant role in various disorders due to their pro-inflammatory functions. Substantially, they are also relevant in neurodegenerative disorders via the delivery of signals for the immune response. However, at the same time, they can aggregate and accelerate the progression of diseases. Natively, S100A8 and S100A9 exist as homo- and heterodimers, but upon aggregation, they form amyloid-like oligomers, fibrils, or amorphous aggregates. In this study, we aimed to elucidate the aggregation propensities of S100A8, S100A9, and their heterodimer calprotectin by investigating aggregation kinetics, secondary structures, and morphologies of the aggregates. For the first time, we followed the *in vitro* aggregation of S100A8, which formed spherical aggregates, unlike the fibrillar structures of S100A9 under the same conditions. The aggregates were sensitive to amyloid-specific ThT and ThS dyes and had a secondary structure composed of β-sheets. Similarly to S100A9, S100A8 protein was stabilized by calcium ions, resulting in aggregation inhibition. Finally, the formation of S100A8 and S100A9 heterodimers stabilized the proteins in the absence of calcium ions and prevented their aggregation.

## Introduction

Calprotectin (CP) is a heterodimer formed from inflammation-associated S100A8 (calgranulin A) and S100A9 (calgranulin B) proteins.^1^ Both are members of the calcium-binding S100 protein family and are greatly expressed in neutrophils, where they potentially account for 45% of all cytosolic proteins.^2^ The S100 proteins are part of a larger EF-hand family, characterized by a helix–loop–helix motif (EF-hand), that binds calcium ions.^3^ Calcium ions stabilize CP and help to form heterotetramer, which is essential for tubulin binding^4^ and protein–protein interactions.^5^ CP functions primarily include triggering an innate immune response and activation of an inflammatory chain reaction.^6^ Secreted CP binds to receptors of advanced glycation end products (RAGE) and Toll-like receptor 4 (TLR4).^7^ Separately, S100A8 and S100A9 can be found as homodimers as well with distinct intracellular and extracellular functions.^8^

As a result of the strong upregulation of S100A8, S100A9, and CP during inflammation, they are frequently associated with the progression of various diseases.^9^ They can be used as biomarkers for monitoring local inflammatory activity in dermatitis,^10^ colorectal cancer,^11^ myocarditis,^12^ COVID-19,^13^ and Alzheimer’s disease.^14^ Considering that neuroinflammation itself is a major risk factor in Alzheimer’s disease progression,^15,16^ S100 proteins can directly participate in neurodegenerative disorder progression.^17^ Foremost, S100 proteins, despite their globular structure, can form amyloids,^18^ insoluble protein aggregates that are hallmark features of many neurodegenerative disorders.^19^ Furthermore, S100B, S100A1, S100A8, S100A9, and S100A12 have been shown to have a role in brain disorders through inhibiting amyloid aggregation, regulating enzyme activity and colocalization with other key proteins associated with neuropathologies.^20−23^

**Figure 1:**
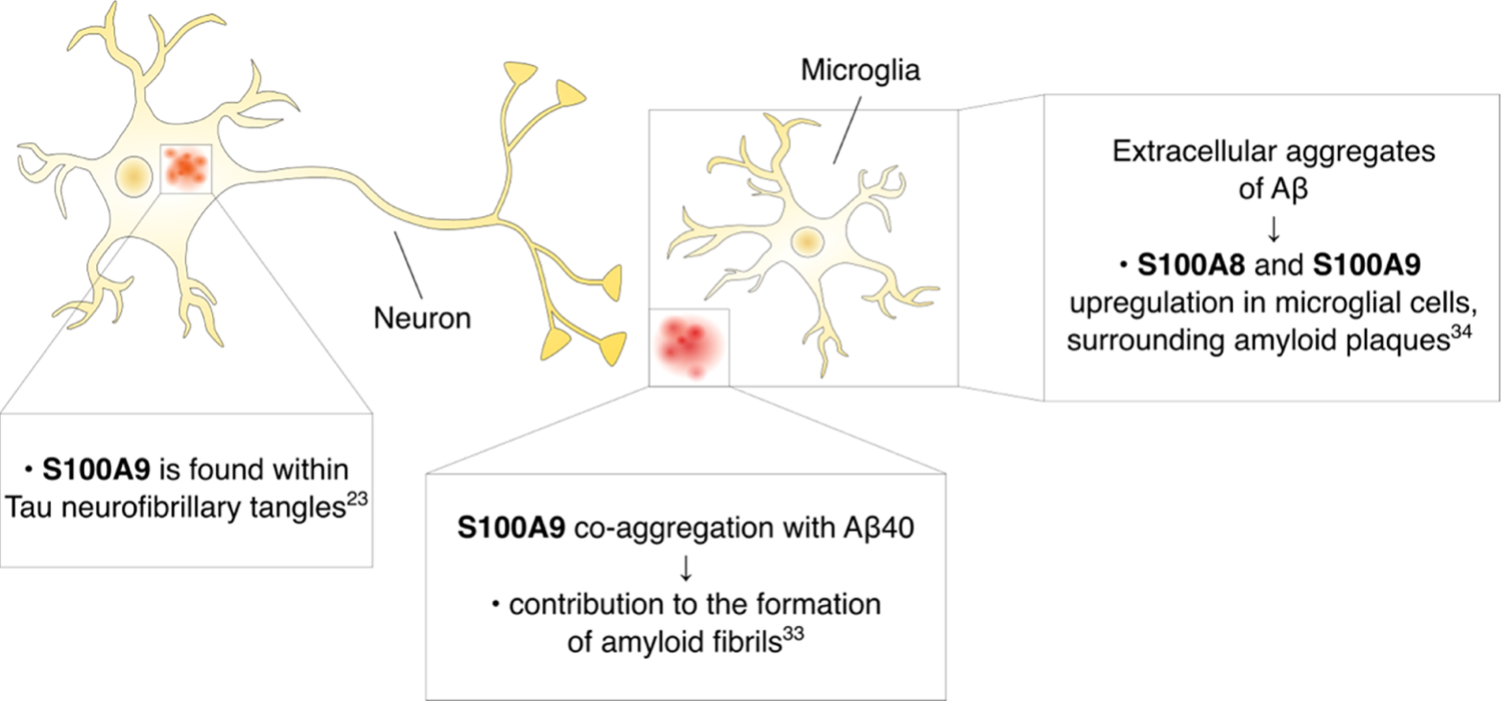
S100A8 and S100A9 play roles in the neurodegeneration. S100A9 was detected in Tau neurofibrillary tangles^23^ and coaggregated with amyloid-β 40 (Aβ40).^33^ Both proteins are upregulated in microglial cells in response to amyloid plaques.^34^

Notably, in the past two decades, S100A8, S100A9, and their heterodimers have been observed to have a significant effect on neurons and microglia during neurodegenerative disorders (Figure 1), and in certain scenarios their amyloid formation has been observed.^22,24^ Taking into account that amyloid aggregation is one of the first indications of Alzheimer’s disease,^25^ it is essential to understand the aggregation of S100A8 and S100A9. In the case of S100A9, considerable knowledge already exists regarding its amyloidogenic properties^26^ and previously published studies demonstrate how S100A9 can affect the aggregation of amyloid-β (Aβ)^27^ and α-synuclein,^28^ main proteins associated with Alzheimer’s and Parkinson’s diseases, respectively.^29,30^ On the other hand, S100A8 aggregates have been only observed in the transgenic mice models,^22^ and CP is only known to form amyloid fibrils in the aging prostate.^31^ Aggregation of S100A8 and S100A9 was also observed in yeast models, where aggregates were nontoxic, but created a large strain of protein quality control system.^32^ Comprehensively, S100A9 aggregation is much more detailed compared with that of S100A8 and CP, although their biological roles are strongly intertwined. Furthermore, it is still unclear how these two proteins affect each other aggregation.

In this study, we aimed to investigate S100A8, S100A9, and CP aggregation and characterize the formed aggregates. For each protein, we performed a Thioflavin T fluorescence assay and observed that S100A8, unlike S100A9, aggregates via different kinetics and is stabilized by calcium ions as well. More importantly, the formation of CP inhibited the aggregation of both proteins. Secondary structure analysis and various microscopic techniques helped identify that S100A8 formed aggregates containing β-sheet structures similar to amyloid fibrils. Altogether, we showed that the formation of CP is an important event in stabilizing S100A8 and S100A9 proteins in the absence of calcium ions.

## Results and Discussion

### S100A8, S100A9, and CP Aggregation Propensities

**Figure 2:**
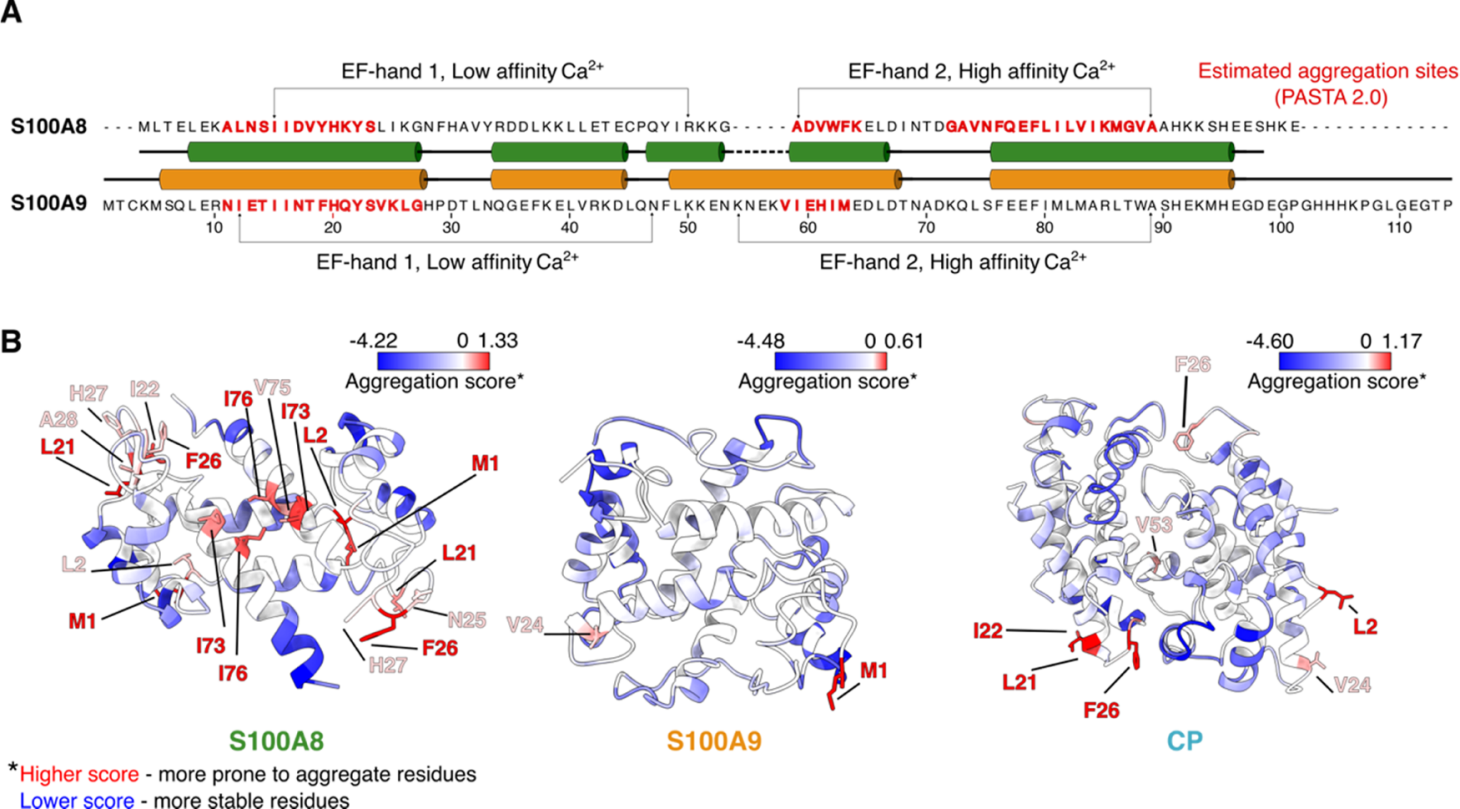
S100A8 and S100A9 amino acid sequence and secondary structure depiction with possible aggregation sites predicted by PASTA 2.0^35^ (A). The aggregation-prone residues identified by Aggrescan3D^37^ using available protein structures (S100A8–1MR8; S100A9–1IRJ, CP-1XK4) (B). The positive scores (red, sticks) indicate high-potential aggregation sites, and negative scores (blue) show more stable residues.

As an initial step for investigating S100A8 and S100A9 aggregation propensities, we evaluated and compared their amino acid sequences by protein aggregation prediction server PASTA 2.0.^35^ It revealed aggregation sites in S100A8 between A8-S20, A51-K56, and G64-A81 and in S100A9 between N11-G27 and V58-M63 residues (Figure 2A). All aggregation sites are part of the EF-hands that bind calcium ions, which matched previous observations.^36^ To examine CP aggregation sites, we used Aggrescan3D Web server,^37^ which performs analysis on the available structures and identifies general aggregation propensities. The currently solved CP structure models are depicted as tetramers, whereas S100A8 and S100A9 are depicted as homodimers. All of them contain metal ions that stabilize their structures,^38^ thereby the comparison between them can only be done in a limited manner. Taking these concerns into account, Aggrescan3D indicated that S100A8 was least stable, with residues in the dimer interface (I73, V75, and I76) and the first EF-hand (L21, I22, N25, F26, H27, and A28) being the most aggregation-prone (Figure 2B). Besides the initial methionine, the S100A9 structure did not display any high-potential aggregation sites. The heterodimer exhibited increased stabilization of the S100A8 monomer, with the least stable residues in the first calcium-binding hand. Considering that these models are only theoretical, we followed up with experiments to investigate calcium ions and CP formation effect on protein stability and aggregation.

### Stability Characterization of S100A8, S100A9, and CP

**Figure 3:**
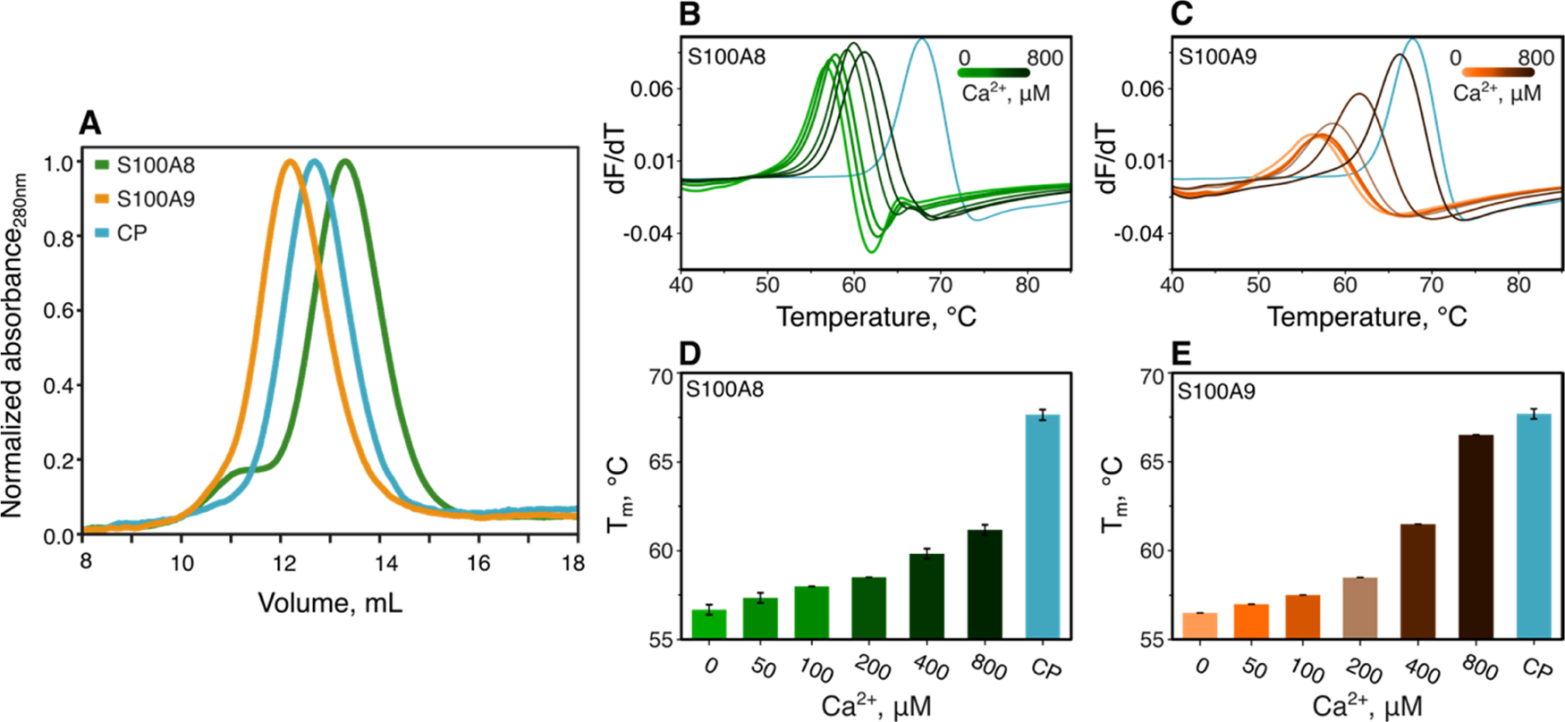
Characterization of the CP formation. Size exclusion chromatography chromatogram of S100A8, S100A9, and CP (A). The first derivative of the normalized DSF fluorescence signal of S100A8 (B) and S100A9 (C) in the absence and presence of calcium ions (0, 50, 100, 200, 400, and 800 μM Ca^2+^). Calculated melting temperatures (using the first derivative) of each protein (D, E).

According to previously established protocol,^38^ we observed that CP formation can be achieved in the absence of calcium, based on elution profiles from size exclusion chromatography. Our results showed that CP eluted between S100A8 and S100A9 homodimers (Figure 3A), matching the expected size of the complex. Complementary to gel filtration, native-PAGE also confirmed heterodimer presence, as the band of CP migrated slower than S100A9 but faster than S100A8 (Figure S1). To identify the potential presence of protein oligomers or aggregates, we performed DLS measurements (Figure S2). Determined S100A8 (2.02 ± 0.1 nm), S100A9 (2.71 ± 0.1 nm), and CP (2.41 ± 0.6) hydrodynamic radius matched with previously established S100 dimers’ radius values ranging from 2.0 to 3.0 nm,^27,38,39^ which might vary due to different buffer conditions and instrumentation. There was also a small amount of larger particles, possibly indicating a higher level of oligomerization or aggregation.

To characterize the stability of S100A8, S100A9, and CP, we monitored protein thermal unfolding using DSF (Figure 3B,C). The melting temperatures of S100A8 and S100A9 homodimers were determined to be 56.7 and 56.5 °C, respectively. However, CP melted at 67.7 °C (Figure 3D,E), indicating a more stable heterodimer complex compared with homodimers. These results mirrored previously observed denaturation temperatures using differential scanning calorimetry.^38^ Furthermore, we investigated the effect of calcium ions on the stability of S100A8 and S100A9 proteins. It was observed that melting temperatures of both proteins increased with the addition of calcium ions (Figure 3B–E), and especially S100A9 showed a better-resolved curve, indicating a more stable conformation of the protein. The presence of calcium ions had an overall smaller effect on S100A8 compared with S100A9, potentially due to disordered S100A9 C-terminal tail,^40^ which can bind additional metal ions. These results also confirmed our bioinformatical prediction that the metal binding loops are essential sites for S100A8 and S100A9 stability and agreed with previously reported nuclear magnetic resonance spectroscopy experiments on S100A9.^41^ While lower protein stability can be an important factor for increased protein aggregation,^42^ it does not correlate with amyloid formation.^43^ Thus, in further experiments, we compared the S100A8, S100A9, and CP abilities to form amyloid aggregates.

### Aggregation Kinetics of S100A8, S100A9, and CP

**Figure 4:**
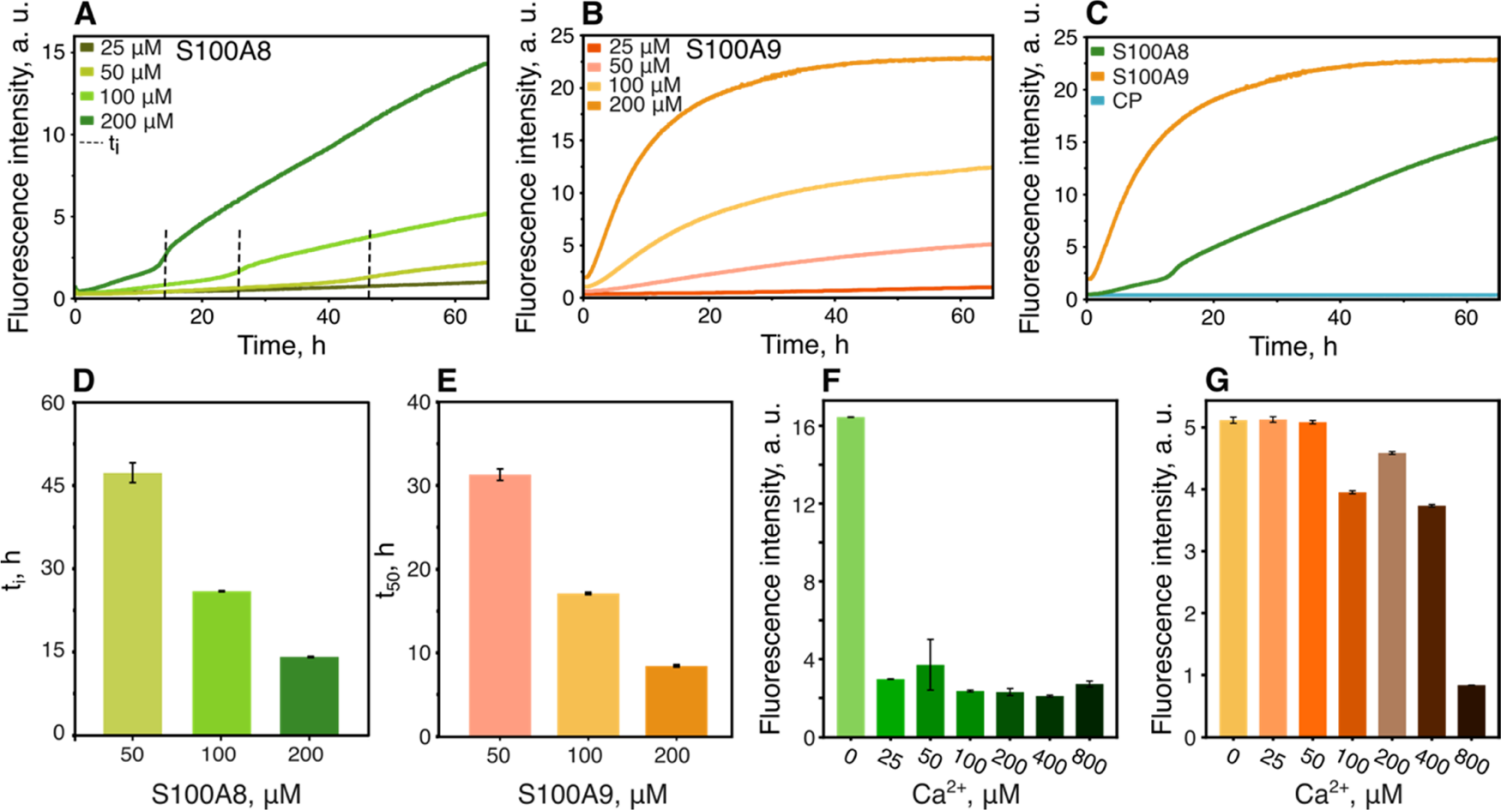
Aggregation kinetics of S100A8 (A), S100A9 (B), and CP (C) followed by ThT fluorescence intensity. The inflection points (t_i_) of S100A8 aggregation curves (D) and half-time values (*t*_50_) of S100A9 aggregation kinetics (E). ThT fluorescence intensity after 70 h of aggregation of S100A8 (F) and S100A9 (G) in the presence of calcium ions (0, 25, 50, 100, 200, 400, and 800 μM Ca^2+^).

Protein aggregation was followed using an amyloid-specific fluorescent ThT dye assay.^44^ Aggregation of both S100A8 and S100A9 started with short or without any initial lag phases (Figure 4A,B), which is atypical for amyloid-forming proteins, that usually follow three-step kinetics (nucleation, elongation, and saturation).^45^ However, such tendencies were observed in oligomeric amyloid species^46^ or worm-like fibrils.^47^ S100A9 aggregation kinetics were already described previously^26^ and correlated with our results as t_50_ was proportional to the S100A9 concentration (Figure 4E). On the other hand, S100A8 aggregation kinetics were not observed before and indicated a different aggregation mechanism compared with S100A9. S100A8 aggregation curves consisted of two phases separated by the inflection point (Figure 4D), which indicates a switch in protein aggregation kinetics. The two-phase aggregation displayed different ThT fluorescence levels, which might be attributed to conformational changes in aggregates or a combination of amorphous and amyloid aggregation happening at the same time.^59^ Although at 37 °C, a 65 h time frame was not enough to monitor the complete aggregation kinetics of S100A8, by increasing the temperature to 42 °C, we were able to observe a second plateau in the same experiment duration (Figure S3A). For further investigation of the S100 protein amyloid nature, we monitored kinetics with the inclusion of 10% preformed aggregates (seeds) of each protein, which resulted in an extended first phase of S100A8 and slightly accelerated S100A9 aggregation (Figure S4). Previously, we concluded that S100A9 aggregation is dominated by the β-sheet formation within the S100A9 molecule and is followed by slow worm-like fibril growth with no fragmentation,^26^ therefore seeding has only a small effect on aggregation. This was also observed with prion protein,^48^ β2-microglobulin,^49^ and transthyretin,^50^ which also formed worm-like fibrils or intermediate amyloidogenic species. Moreover, the addition of calcium ions inhibited the aggregation of both proteins (Figures 4F,G and S5A,B), albeit in a divergent manner. After 70 h of aggregation, the addition of calcium completely arrested the aggregation of S100A8, with an even lower concentration calcium range (1.25–20 μM (Figure S5C,D)) still slowing down kinetics. On the other hand, S100A9 required a saturated solution to curb its amyloid fibril formation.

Finally, when we tested CP, we did not observe any ThT fluorescence increase (Figure 4C) or differences in another amyloid-specific dye, Congo red (CR), absorbance spectra^51^ (Figure S6), which implies inhibited amyloid aggregation. On the contrary, CR spectra of S100A9 aggregates showed typical absorbance peak red shift at 500 nm and the appearance of a peak at 530 nm. S100A8 aggregates, unlike ThT fluorescence, displayed minor changes in CR, which can happen in the case of oligomers that weakly bind CR.^52^

### Secondary Structure of S100A8, S100A9, and CP Aggregates

**Figure 5:**
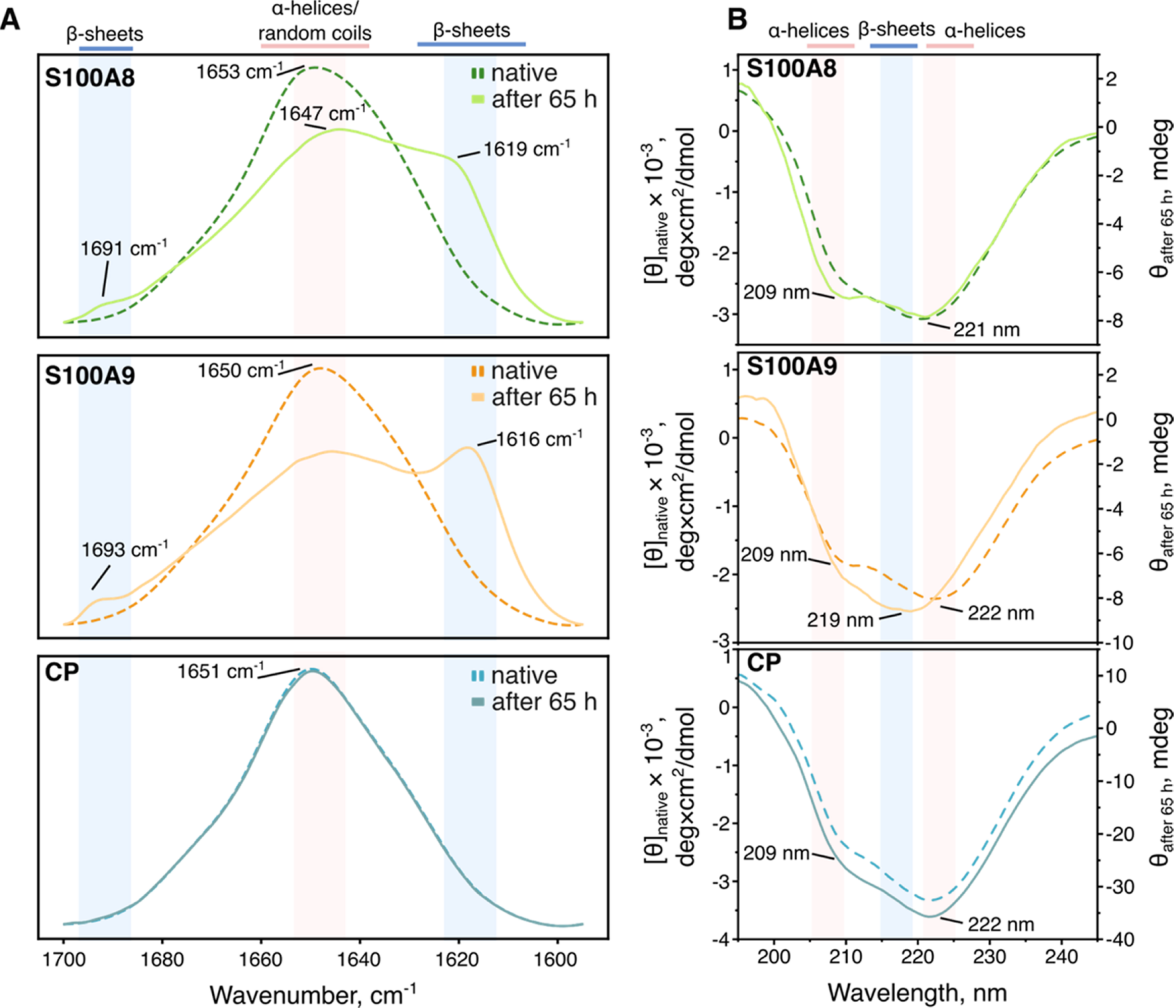
FTIR (A) and CD spectra (B) of native S100A8, S100A9, and CP proteins and after 65 h of aggregation at 37 °C.

The FTIR spectra of native S100A8, S100A9, and CP proteins (Figure 5A) had maxima at 1653, 1650, and 1651 cm^–1^, respectively, indicating α-helical structures.^62^ After aggregation of the proteins, S100A8 and S100A9 propensity of helical structures was reduced, and bands at 1619 cm^–1^ (S100A8) and 1616 cm^–1^ (S100A9) were observed, which correspond to the formation of β-sheets.^53^ Additionally, for both proteins’ clear maxima at 1691 and 1693 cm^–1^ were noticed, which potentially imply antiparallel arrangements of the β-sheets.^53^ The appearance of β-sheet structures in FTIR spectra might suggest various aggregate types: from amyloid fibrils and oligomers to folding or amorphous aggregates.^54^ In the case of CP, no changes in secondary structure were detected, as the FTIR spectrum of incubated protein remained the same as native.

As supplementary data for FTIR, CD spectra were recorded for each sample (Figure 5B). All native samples indicated α-helical fold, with minima at 221/222 nm. The aggregated spectrum of S100A8 displayed a stronger minimum at 209 nm, which would suggest changes in the secondary structure; however, we have not observed β-sheets in contradiction to our FTIR data (Figure 5A). Since in FTIR measurements aggregates were centrifuged, washed, and concentrated in heavy water and in CD the aggregate solution was measured without any additional steps, a plausible explanation is that the S100A8 protein does not completely aggregate. This was confirmed by SDS-PAGE analysis of aggregate solution supernatants (Figure S7), as there was almost no soluble S100A9, but the S100A8 sample still showed a strong band of native protein. The CD spectrum of S100A9 aggregates exhibited a typical β-sheet structure fold,^55^ confirming that it fully assembled into aggregates. Finally, no changes were observed in the CP secondary structures, indicating that no aggregation occurred.

### Morphology of S100A8, S100A9, and CP Aggregates

**Figure 6:**
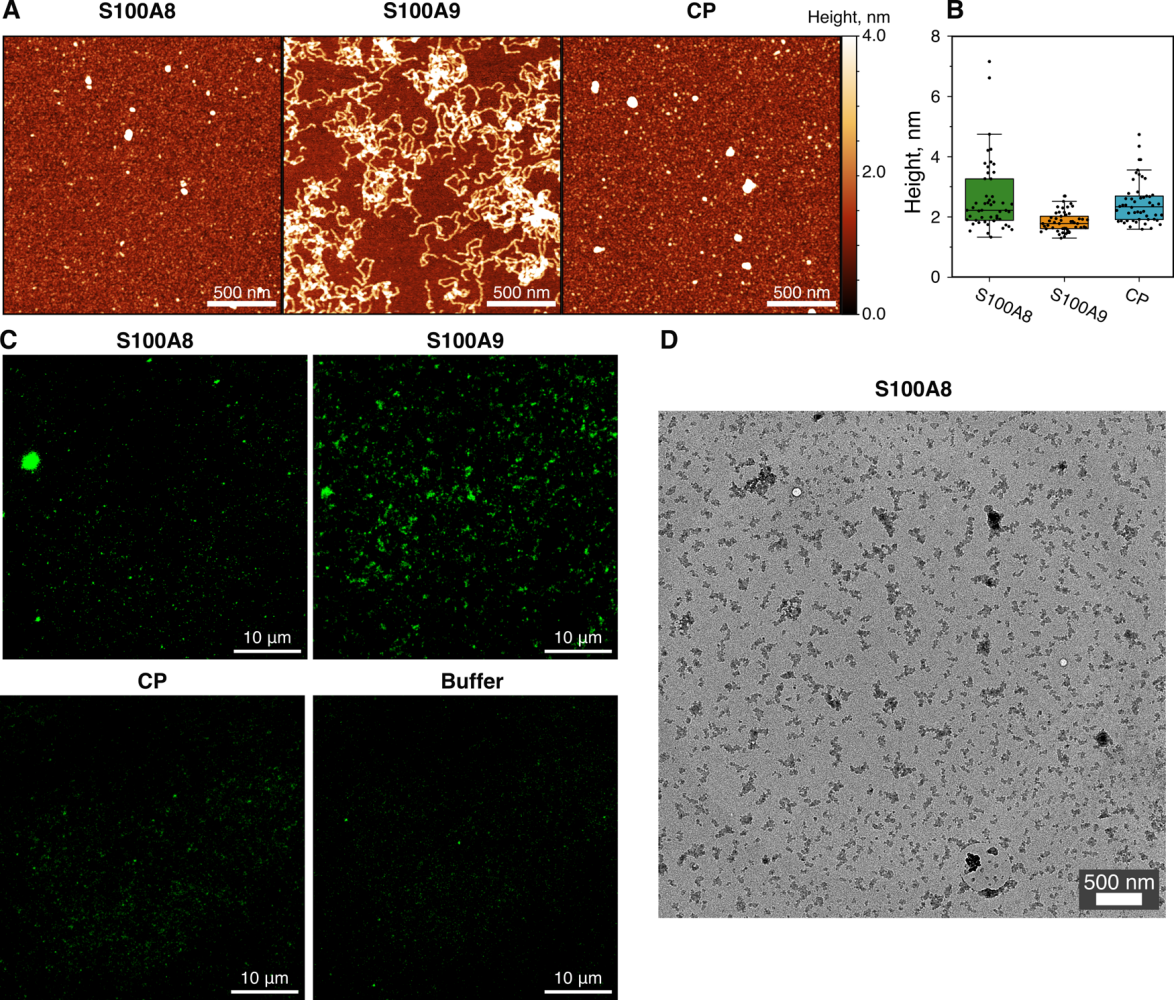
AFM images (A) of S100A8, S100A9, and CP after 65 h of aggregation at 37 °C (scale bar, 500 nm). Aggregates height distribution (B) with box plots indicating the interquartile range and error bars are for one standard deviation (sample size, 50). Fluorescence microscopy images (C) of S100A8, S100A9, CP aggregates, and buffer stained with ThS (scale bar, 10 μm). Transmission electron microscopy image (D) of S100A8 aggregates stained with uranyl acetate (scale bar, 500 nm).

We visualized S100A8, S100A9, and CP aggregates using AFM (Figure 6A and S3B). S100A8 formed spherical oligomers and aggregates with varying heights with an average of 4.7 nm (Figure 6B). Although we attempted to separate S100A8 aggregates by centrifugation to distinguish them from the residual unaggregated protein, large structures were still observed in both samples (Figure S8). S100A9 assembled into worm-like fibrils with an average of 1.8 nm height, which was already described in previous studies.^26,27^ CP solution resembled the S100A8 aggregates, as AFM lacked the resolution to identify differences between the two samples (Figure S9). We did not observe any fibrils formed by CP under our experimental conditions (pH 7.4). However, CP has been shown to assemble into amyloid fibrils *in vitro* at pH 2.5.^18^ At acidic conditions, S100 proteins dissociate from dimers into monomers;^56^ therefore, it is highly likely that proteins aggregated as individual monomers in those conditions, but the nascent interactions cannot be ruled out. For S100A8 and CP, we also incubated samples for 4 weeks, to observe possible further changes (Figure S10). After such a long incubation, the aggregates for S100A8 remained similar, but for CP we observed S100A9-like fibrils, possibly due to dissociation of the heterodimer.

Overall, after 65 h of aggregation, we observed large oligomers in the CP solution that have been identified previously,^31^ but ThT fluorescence assay and secondary structure analysis suggested that they are not amyloid-like. This could be explained by the fact that S100 proteins can assemble into larger native multimeric assemblies.^36^ Nevertheless, we additionally investigated aggregates by fluorescence microscopy using amyloid-specific ThS dye^57^ (Figures 6C and S11). In the S100A9 sample, we observed a substantial number of aggregates, in contrast to S100A8, which had fewer clusters of aggregates. This further indicated that the protein does not completely aggregate. However, since the formed aggregates were sensitive to amyloid-specific dye, they might belong to pathogenic protein species.^58^ On the other hand, CP resembled a buffer sample, where only the background fluorescence of ThS was noticeable. Finally, we imaged S100A8 aggregates with a transmission electron microscope (Figures 6D and S12) and did not identify any fibral structures, but observed particles that looked similar to previously observed Immunoglobulin G aggregates or oligomeric complexes.^59^ However, more in-depth characterization of S100A8 aggregates would require cryogenic electron microscopy or nuclear magnetic resonance spectroscopy to fully understand their origin.

## Conclusions

Neuroinflammation is one of the major factors that fuel neurodegenerative disorders through the activation of microglia^16^ via the accumulation of amyloid deposits.^60^ Hence, the pro-inflammatory proteins become prime research points since they are one of the first molecules responding to the inflammation. CP is recognized as a critical biomarker for various inflammatory disorders^61^ and, in the context of neurodegeneration, both S100A8 and S100A9 are upregulated in the brain, resulting in the increased amount of CP in the cerebrospinal fluid during Alzheimer’s disease.^22,23^ Furthermore, the fecal CP increase has been identified in 73% of Alzheimer’s disease patients,^62^ demonstrating that CP circulation in patients is widely spread. Combining the amyloid aggregation of CP protein^31^ and its proliferation, it is essential to understand the stability of CP and its individual components.

In our study, we showed that both S100A8 and S100A9 homodimers rapidly assembled into aggregates, and the addition of calcium ions or the formation of CP completely inhibited this process. S100A8, unlike S100A9, did not form fibrils but assembled into oligomers and spherical aggregates that bind amyloid-specific ThT and ThS dyes. As oligomers and early species of amyloids are the driving force of neurodegeneration,^63^ S100A8 aggregates raise further questions about their potential in neuropathies. Although previous study indicated their presence in the hippocampus and their positive feedback loop with Aβ,^22^ the role of S100A8 aggregates in neurodegeneration is still poorly understood. Furthermore, the cytoplasmic calcium concentration is maintained low at 100 nM in the cells,^64^ which is below the level needed to slow the aggregation of both proteins, according to our data. Considering that the calcium affinity of S100 proteins’ two EF-hands is 10–50 μM and 200–500 μM,^65^ they could only bind calcium in the influx of calcium ions^66^ and, during the cell resting state, exist in apo-states, that are prone to aggregation. In Alzheimer’s disease, calcium dysregulation has been observed due to cell membrane damage, leading to increased intracellular calcium concentrations.^67^ Therefore, aggregation of S100 proteins might happen before the onset of the disorder and due to prolonged inflammation.

CP is a dominant form of S100A8 and S100A9 *in vivo* regarding their biological role,^1^ and in addition to that, CP might act as a safety measure in preventing aggregation of homodimers. With increased and uneven S100A8 and S100A9 expression during inflammation, CP formation could be impaired, leading to protein aggregation and the advancement of disease. Targeting the S100A8 and S100A9 interactions can be a potential therapeutic approach to limit the damage caused by inflammation.

## Methods

### Aggregation-Sites Analysis

Aggregation protein sites were determined using PASTA 2.0^35^ and Aggrescan3D^37^ using default parameters. Generated results of web servers can be found in the Supplementary Data.

### Cloning

The S100A8 and S100A9 genes and the SUMO-tag were amplified and fused using standard PCR methods. The products were inserted into a pET28a(−) vector via NdeI and *Bam*HI restriction sites by standard cloning techniques yielding SUMO-S100A8 and SUMO-S100A9 constructs fused to an amino-terminal His_6_ tag. Plasmids and primers used in this study can be found in Table S1.

### Protein Expression and Purification

The pDS98 and pDS99 plasmids containing His_6_-SUMO-S100A8 and His_6_-SUMO-S100A9 were chemically transformed into One Shot *E. coli* BL21(DE3) cells (Thermo Scientific). The bacterial cells were grown in ZYM-5052 autoinducing growth medium^68^ supplemented with kanamycin (50 μg/mL) at 37 °C, 220 rpm until optical density measured at a wavelength of 600 nm (OD_600_) of 0.6–0.8 was reached. The temperature was lowered to 25 °C and the incubation was continued for an additional 18 h. The cells were harvested by centrifugation (20 min, 6000*g*, 4 °C) and resuspended in 25 mM HEPES (pH 7.5) buffer containing 1 M NaCl, 10 mM imidazole, lysozyme, and 1 mM phenylmethylsulfonyl fluoride. The suspension was lysed by sonication for 30 min, using 40% power (cycles of 10 s on, 30 s off) with a Sonopuls (Bandelin) homogenizer. The lysate was incubated with the universal nuclease for cell lysis (Pierce, Thermo Scientific) and 1 mM MgCl_2_ for 20 min on ice. To remove cellular debris, the sample was centrifuged (45 min, 18,000*g*, 4 °C).

After the filtration using a 0.45 μm pore size filter, the sample was applied to a Ni^2+^ Sepharose 6 Fast Flow (Cytiva) loaded gravity column, previously equilibrated with 25 mM HEPES (pH 7.5) buffer containing 1 M NaCl and 10 mM imidazole. Stepwise elution was performed using 25 mM HEPES (pH 7.5) buffers containing 1 M NaCl, 10 mM, 75 mM, and 300 mM imidazole. Fractions containing the His_6_-SUMO-tagged proteins were dialyzed at 4 °C for 2 h against 20 mM Tris-HCl (pH 7.5) buffer. Human Sentrin-specific protease 1 (SENP1) catalytic domain (derived from pET28a-HsSENP1 (a gift from Jorge Eduardo Azevedo, Addgene plasmid #71465)) was added to the samples for His_6_-SUMO-tag proteolysis, and dialysis was continued for an additional 18 h in fresh 20 mM Tris-HCl (pH 7.5) buffer. The samples were centrifuged (20 min, 18,000*g*, 4 °C) and the supernatants were applied to a Ni^2+^ column. The flow-through was collected and concentrated using Amicon centrifugal filters (10 kDa Molecular weight cutoff (MWCO), Merck Millipore).

Prior to size exclusion chromatography, the protein sample was incubated with 1 mM TCEP and 1 mM EDTA for 1 h on ice and loaded on the HiLoad 26/600 column Superdex 75 prep grade column (Cytiva), precalibrated with 50 mM HEPES buffer (pH 7.4). The purity of collected fractions was assessed by SDS-PAGE and protein was concentrated using Amicon centrifugal filters (10 kDa MWCO, Merck Millipore). The purified samples of S100A8 and S100A9 were stored at −80 °C. Before each use, protein samples were centrifuged (10 min, 16,900*g*, 4 °C). The size exclusion chromatography profiles and comparisons between different purification batches can be found in the supplement (Figure S13).

The CP was prepared similarly as described previously.^38^ The formation of the S100A8/S100A9 complex included equimolar mixing of purified recombinant S100A8 and S100A9 proteins. The 0.1 M glycine buffer (pH 2.0) was added to a final 20 mL volume, and the pH was adjusted to 2.0–2.5 by adding hydrochloric acid. The denaturation of both proteins’ homodimers in acidic pH^56^ was conducted by incubation at room temperature with stirring for 1 h. Heterodimer formation was achieved by stepwise dialysis at 4 °C against 20 mM Tris-HCl (pH 8.5) buffer containing 1 mM EDTA and 1 mM DTT. The dialyzed sample was centrifuged (20 min, 18,000*g*, 4 °C) and filtered using a 0.45 μm pore size filter.

Anion exchange chromatography was performed using a HiTrap Q HP (Cytiva) column. The column was washed with 20 mM Tris-HCl (pH 8.5) buffer supplemented with 1 mM EDTA and 1 mM DTT. Step elution was achieved using 30, 40, 50, and 100% gradients of the same composition buffer containing 500 mM NaCl. 30% fraction contained equal amounts of S100A8 and S100A9, as detected by SDS-PAGE.

CP formation was assessed by size exclusion chromatography (Tricorn 10/300 column (Cytiva)), packed with Superdex 75 prep grade resin (Cytiva) and native-PAGE. The column was precalibrated with 50 mM HEPES buffer (pH 7.4) containing 1 mM EDTA and 1 mM TCEP. The collected fractions of the heterodimer were concentrated using Amicon centrifugal filters (10 kDa MWCO, Merck Millipore), and the concentration of dimer was determined by UV absorption at 280 nm using an extinction coefficient (ϵ_280_) of 18,450 M^–1^cm^–1^. The purified sample of CP was stored at −80 °C. Before each use, the protein sample was centrifuged (10 min, 16,900*g*, 4 °C).

### Dynamic Light Scattering (DLS)

DLS of protein samples (100 μM S100A8, S100A9, and 50 μM CP; 50 mM HEPES pH 7.4) were measured with the Zetasizer μV (Malvern panalytical), equipped with an 830 nm laser, at 8 °C. The hydrodynamic radius *R*_h_ was calculated with Raynals tool^69^ using the following values: wavelength = 830 nm, scattering angle = 90°, temperature = 8 °C, refractive Index = 1.33, and viscosity = 0.001387 Pa·s.

### Differential Scanning Fluorimetry (DSF)

100 μM S100A8, S100A9, and 50 μM CP in 50 mM HEPES (pH 7.4) buffer containing 100 μM 1,8-anilinonaphthalenesulfonate (ANS), 1 mM TCEP, and different (0, 50, 100, 200, 400, and 800 μM) CaCl_2_ concentrations were used to perform DSF experiments. Samples without Ca^2+^ additionally included 1 mM EDTA. Protein unfolding was monitored with a Rotor-Gene Q instrument (QIAGEN) using the blue channel (excitation, 365 ± 20 nm; detection, 460 ± 20 nm). Constant heating was applied at a rate of 1 °C/min from 25 to 99 °C. The data was normalized and analyzed using MoltenProt software.^70,71^ Thermal melting temperatures (*T*_m_) were determined using the first derivative in the temperature range of 40–80 °C.

### Thioflavin T (ThT) Fluorescence Assay

ThT fluorescence assay was conducted using targeted protein concentrations (25, 50, 100, and 200 μM S100A8, S100A9, 100 μM CP) in 50 mM HEPES (pH 7.4) buffer containing 1 mM TCEP, 50 μM Thioflavin T (ThT), and different (0, 25, 50, 100, 200, 400, and 800 μM) CaCl_2_ concentrations. Samples without Ca^2+^ additionally included 1 mM EDTA. 100 μL of the sample for each well was distributed to a 96-well nonbinding half area plate (Corning). For seeding experiments, 10 μM preformed aggregates were added to 100 μM S100A8 or S100A9 protein solution. Aggregation kinetics were monitored using a ClarioStar Plus plate reader in enhanced dynamic range mode. ThT fluorescence emission intensity (440 nm excitation and 480 nm emission wavelengths) was scanned every 5 min at 37 °C with 5 s orbital shaking before measurement. The half-time values (t50) were obtained by fitting kinetics to the Hill equation as described previously^72,73^ and inflection (t_i_) points were calculated from the first derivative of aggregation curves.

### Congo Red (CR) Absorbance Assay

For the CR assay, 10 μM (S100A8, S100A9) or 5 μM (CP) protein samples (before and after 65 h aggregation at 37 °C) containing 20 μM CR were prepared. Three CR absorbance spectra for each sample were recorded from 200 to 800 nm using a Shimadzu UV-1800 and averaged. The spectra analysis was performed using Quasar.^74^

### Fourier Transform Infrared (FTIR) Spectroscopy

After 65 h of aggregation at 37 °C, samples were removed from the aggregation reaction kinetics plate and were used for the preparation of FTIR measurements (280 μL of each sample). The aggregated samples of S100A8 and S100A9 were centrifuged at 16,900*g* for 30 min, after which the supernatant was removed and replaced with 300 μL of D_2_O supplemented with 500 mM NaCl (addition of NaCl may improve fibril sedimentation).^75^ The centrifugation and resuspension procedure was repeated four times. After the final step, the aggregate pellet was resuspended into 100 μL of D_2_O containing 500 mM NaCl.

CP samples after the aggregation reaction, as well as samples of native S100A8, S100A9, and CP, were prepared using 10 kDa MWCO Pierce protein concentrators (Thermo Scientific). The buffer replacement with D_2_O was achieved by centrifugation at 14,000*g* for 7 min, after which 300 μL of D_2_O was supplemented with 500 mM NaCl. The procedure was repeated eight times. After the final step, the samples were concentrated to a final volume of 100 μL.

FTIR spectra were acquired using an Invenio S FTIR spectrometer (Bruker), equipped with a liquid-nitrogen-cooled mercury cadmium telluride detector, at room temperature and constant dry-air purging. For all measurements, CaF_2_ transmission windows and 0.05 mm Teflon spacers were used. For every sample, 256 interferograms of 2 cm^–1^ resolution were recorded and averaged. D_2_O containing 500 mM NaCl and water vapor spectra were subtracted from each sample spectrum, followed by baseline correction and normalization to the same 1600–1700 cm^–1^ wavenumber range. All data processing was done using GRAMS software.

### Far-UV Circular Dichroism (CD) Spectroscopy

200 μM S100A8, S100A9, and 100 μM CP in 50 mM HEPES (pH 7.4) buffer containing 1 mM EDTA and 1 mM TCEP were used to measure CD spectra. Aggregation of each sample was conducted in a test tube at 37 °C for 65 h. 60 μL of samples of aggregated proteins and native proteins of the same concentration were placed in a 0.1 mm path length quartz cuvette. The CD spectra were measured using a J-815 spectropolarimeter (Jasco). For each sample, five spectra between 190 and 260 nm (every 0.5 nm) were recorded and averaged. 50 mM HEPES (pH 7.4) containing 1 mM EDTA and 1 mM TCEP spectrum was subtracted from each sample spectrum. Additionally, spectra were smoothed using the moving averaging function (rectangular, interval = 7). All data processing was performed using Spectragryph v1.2.16.1 software.

### Atomic Force Microscopy (AFM)

The surface of freshly cleaved mica was modified with 30 μL of 0.5% (% v/v) (3-aminopropyl)triethoxysilane (APTES). After incubation at room temperature for 5 min, APTES was gently washed using 1 mL of water, and the surface of the mica was dried using airflow. Thirty microliters of the protein sample was used to absorb for 5 min on mica, and washing and drying steps were repeated. For S100A8 pellet and supernatant samples, the aggregate solution was centrifuged at 16,900*g* for 15 min, and the supernatant was separated from the pellet. The aggregates were resuspended in 50 mM HEPES (pH 7.4) buffer containing 1 mM EDTA and 1 mM TCEP. AFM imaging was performed using a Dimension Icon (Bruker) atomic force microscope operating in tapping-in-air mode with aluminum-coated silicon tips (RTESPA-300, Bruker). AFM images were analyzed using Gwyddion 2.5.5. software.^76^ The cross-sectional height of aggregates was determined from line profiles, which were fitted using the Gaussian function.

### Fluorescence Microscopy

Glass coverslips (17244914, Fisher Scientific), used for fluorescence imaging of amyloid aggregates, were put into a Hellendahl-type staining jar and cleaned in the following sequence: rinsed with ultrapure deionized water, immersed in 1% (w/v) Alcojet (1404–1, Alconox) detergent aqueous solution, and incubated in an ultrasonic cleaner for 5 min, rinsed with ultrapure deionized water two times, soaked in 1 M NaOH aqueous solution for 1 h, rinsed with ultrapure deionized water two times, rinsed with 2-propanol (CP41.8, Carl Roth), then left on the laboratory table for 10 min to dry, and, ultimately, burned in a laboratory oven at 120 °C for 20 min.

After treating one of the cleaned glass coverslips with an air plasma (∼260 mTorr, highest power setting, ZEPTO-W6, Diener electronic) for 5 min, such a coverslip was assembled into a flow cell by attaching it to a six-channel plastic slide (80606, Ibidi) via a double-sided sticky tape (3 M, 9088–200) spacer. The selected channel of this flow cell was filled with 200 μL of 0.01% (w/v) poly-l-lysine (A-005-M, Merck), incubated for 3 min, and then washed with 600 μL of buffer (50 mM HEPES, pH 7.4). 100 μL of 5 μM protein aggregates, prestained using amyloid-specific ThS dye (1 μM final concentration), was injected into the channel and incubated for at least 3 min before imaging. After the channel was flushed with 400 μL of buffer, fluorescence imaging was performed. For each sample, separate channels of such a flow cell were used for their imaging with an identical sample preparation procedure as described above.

Glass surface-immobilized protein aggregates were visualized by employing our custom-built miEye microscopy system capable of performing single-molecule fluorescence microscopy and super-resolution imaging.^77^ For the measurements in this study, the microscope was set in a single-mode fiber-based excitation scheme with a triple-line beam splitter ZT405/514/647rpc-UF2 (Analysentechnik) inserted in the microscope’s body. The samples were illuminated in total internal reflection fluorescence (TIRF) mode using a 405 nm wavelength laser for ThS excitation. The light emitted by this dye was passed through a 525/45 band-pass filter and collected using an industrial CMOS camera (Alvium 1800 C-511m, Allied Vision Technologies) with its exposure time set to 100 ms.

### Transmission Electron Microscopy (TEM)

Five microliter portion of prepared 100 μM S100A8 aggregate solution was applied to the glow-discharged 300 mesh copper grids (Agar Scientific) for 1 min. After the excess fluid was removed with filter paper, the grid was negatively stained with 5 μL of 2% (w/v) uranyl acetate for 1 min, followed by three 1 min washes with 5 μL of water. All TEM images were acquired on a Talos 120C (Thermo Fisher) microscope operating at 120 kV and equipped with a 4k × 4k Ceta CMOS Camera.

## Supporting Information Available

The raw data (fluorescence kinetics, CD/FTIR, microscope images, DLS) are available on Mendeley Data repository: 0.17632/j8bmnv4vzp.1. All other relevant data are available from the corresponding author upon reasonable request (PDF)
