# Supplementary material for: Formation of Calprotectin Inhibits Amyloid Aggregation of S100A8 and S100A9 Proteins
Source: ACS Chem Neurosci. 2024 Apr 18;15(9):1915–25. doi: 10.1021/acschemneuro.4c00093 (PMC11066842; doi:10.1021/acschemneuro.4c00093)
Supplement: Supplementary file 1 — cn4c00093_si_001.pdf [file cn4c00093_si_001.pdf]

Supporting information

for

Formation of calprotectin inhibits amyloid aggregation of S100A8 and S100A9 proteins

Ieva Baronaitė[1], Darius Šulskis[1], Aurimas Kopūstas[1, 2], Marijonas Tutkus[1, 2], Vytautas Smirnovas[1]

[1] Institute of Biotechnology, Life Sciences Center, Vilnius University, LT-10257 Vilnius, Lithuania

[2] Department of Molecular Compound Physics, Center for Physical Sciences and Technology, LT-10257 Vilnius, Lithuania

*Correspondence should be addressed to

Email: darius.sulskis@gmc.vu.lt

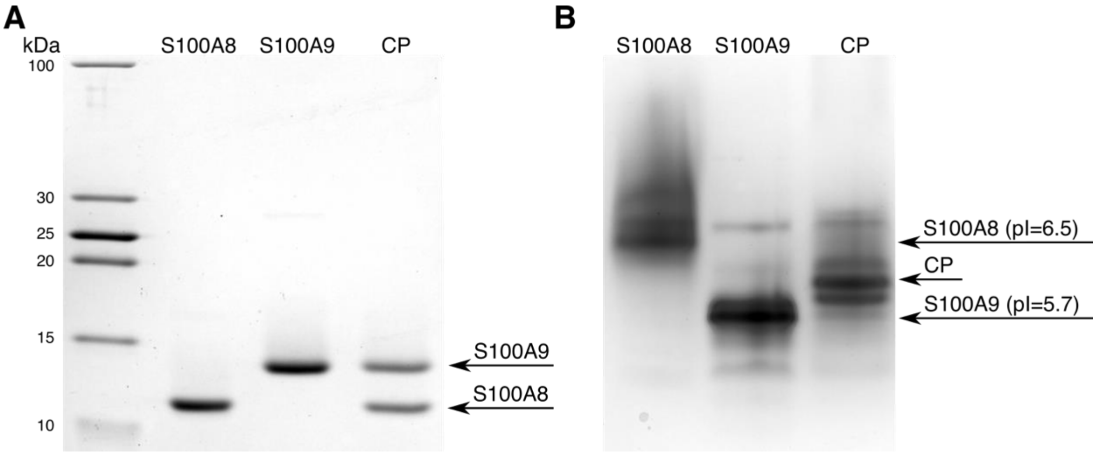

Figure S1. SDS-PAGE (**A**) and native-PAGE (**B**) of S100A8, S100A9 and CP.

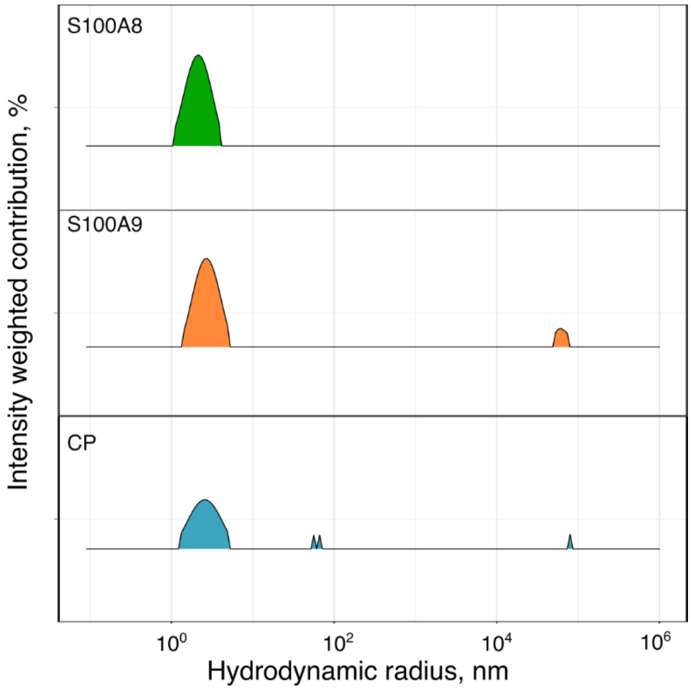

Figure S2. DLS analysis of S100A8, S100A9 and CP proteins size distributions (average of 7 scans with 10 reads).

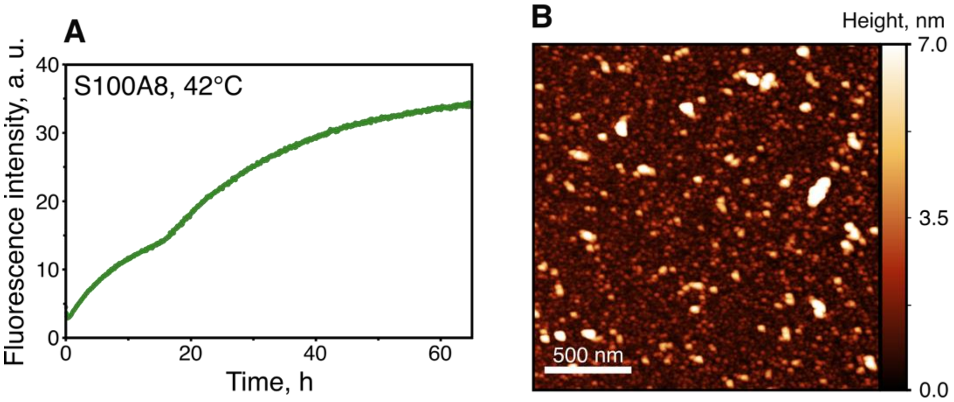

Figure S3. Aggregation kinetics (**A)** of 100 μM S100A8 at 42°C and AFM image (**B**) of aggregates at the endpoint (scale bar, 500 nm).

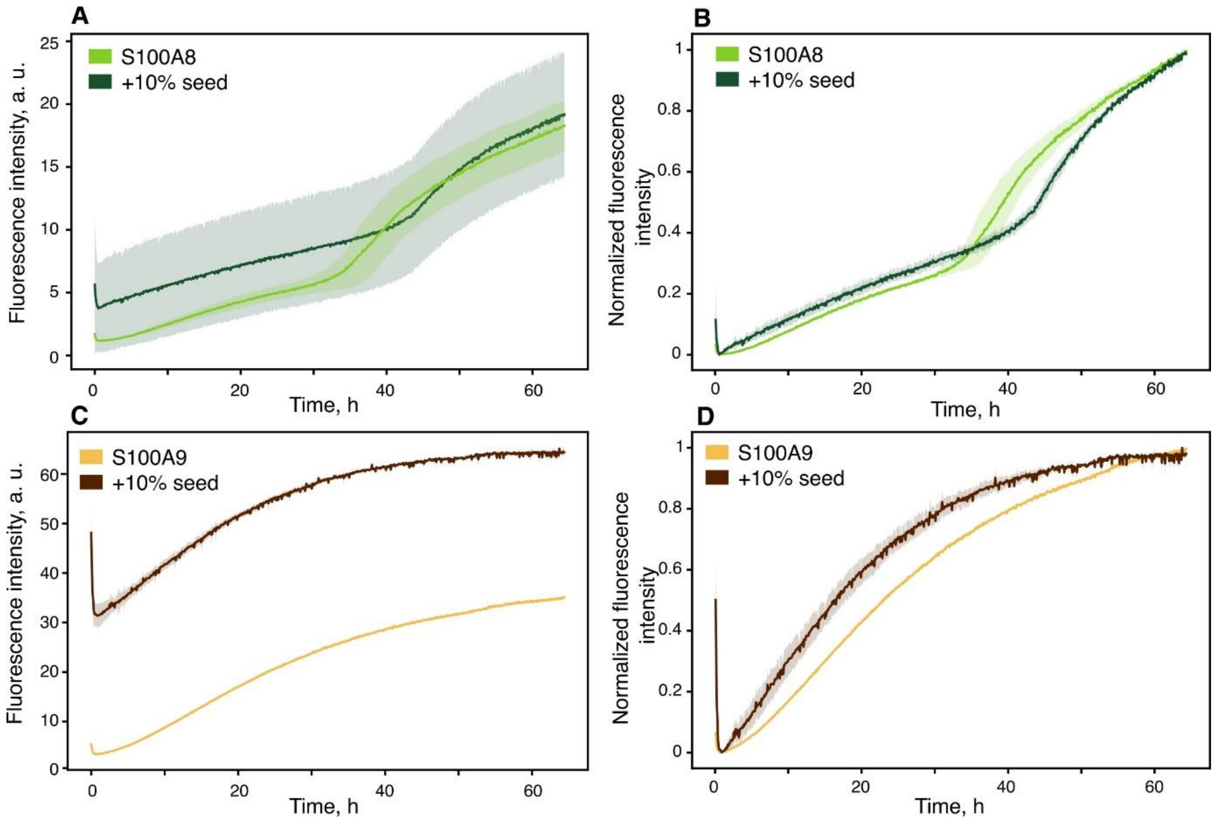

Figure S4. Raw and normalized ThT fluorescence aggregation kinetics of 100 μM S100A8 (**A, B**), 100 μM S100A9 (**C, D**) in the presence of 10% preformed aggregates (seed) of each protein, respectively.

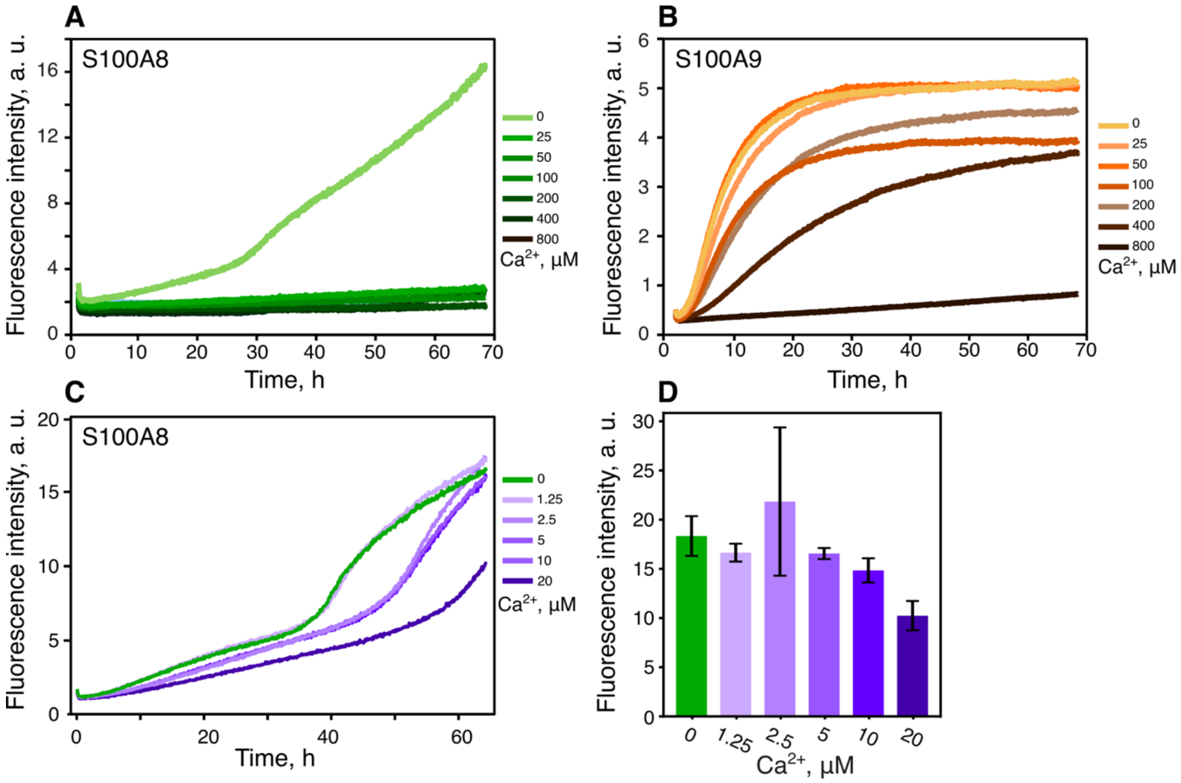

Figure S5. Aggregation kinetics of 100 μM S100A8 (**A**), 100 μM S100A9 (**B**) in the presence of calcium ions (0, 25, 50, 100, 200, 400, 800 μM Ca^{2+}). Aggregation kinetics of 100 μM S100A8 (**C**) and endpoint fluorescence (**D**) in low calcium concentrations (0, 1.25, 2.5, 5, 10, 20 μM Ca^{2+}). All kinetics were followed by ThT fluorescence intensity.

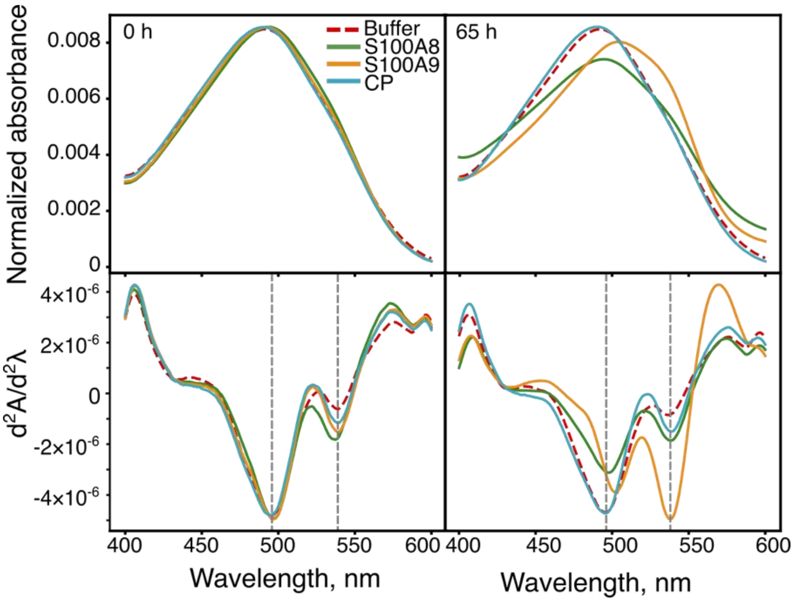

Figure S6. The Congo red dye absorbance spectra and second derivative in the presence of buffer solution, S100A8, S100A9 and CP at 0 h and 65 h after aggregation.

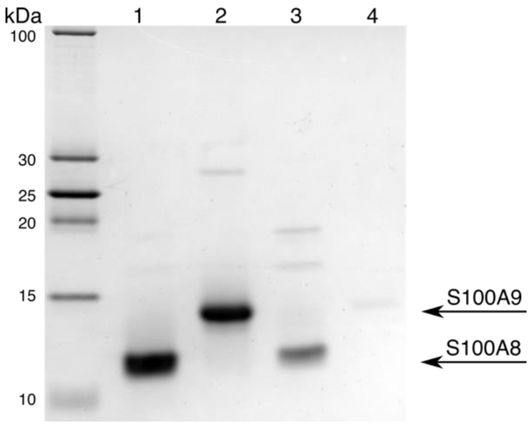

Figure S7. SDS-PAGE samples before aggregation: S100A8 (lane 1), S100A9 (lane 2). Supernatants after 65 h of aggregation: S100A8 (lane 3), S100A9 (lane 4).

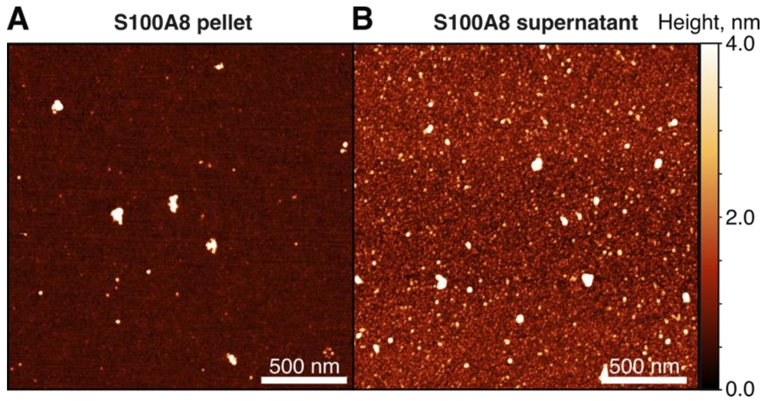

Figure S8. S100A8 pellet (**A**) and supernatant (**B**) AFM images after 65 h of aggregation (scale bar, 500 nm).

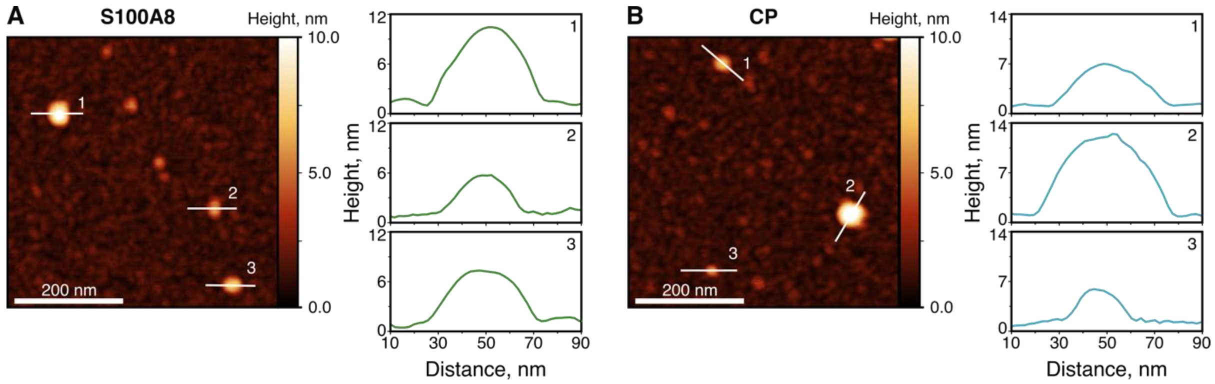

Figure S9. The height profiles of S100A8 (**A**) and CP (**B**) particles from AFM images (scale bar, 200 nm).

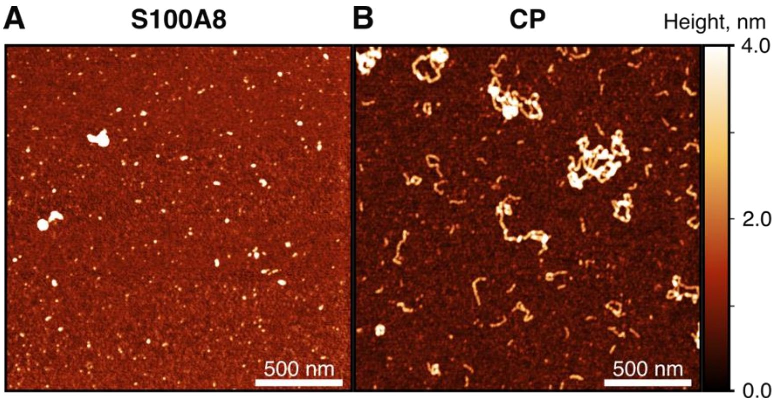

Figure S10. S100A8 (**A**) and CP (**B**) AFM images after 4 weeks of aggregation (scale bar, 500 nm).

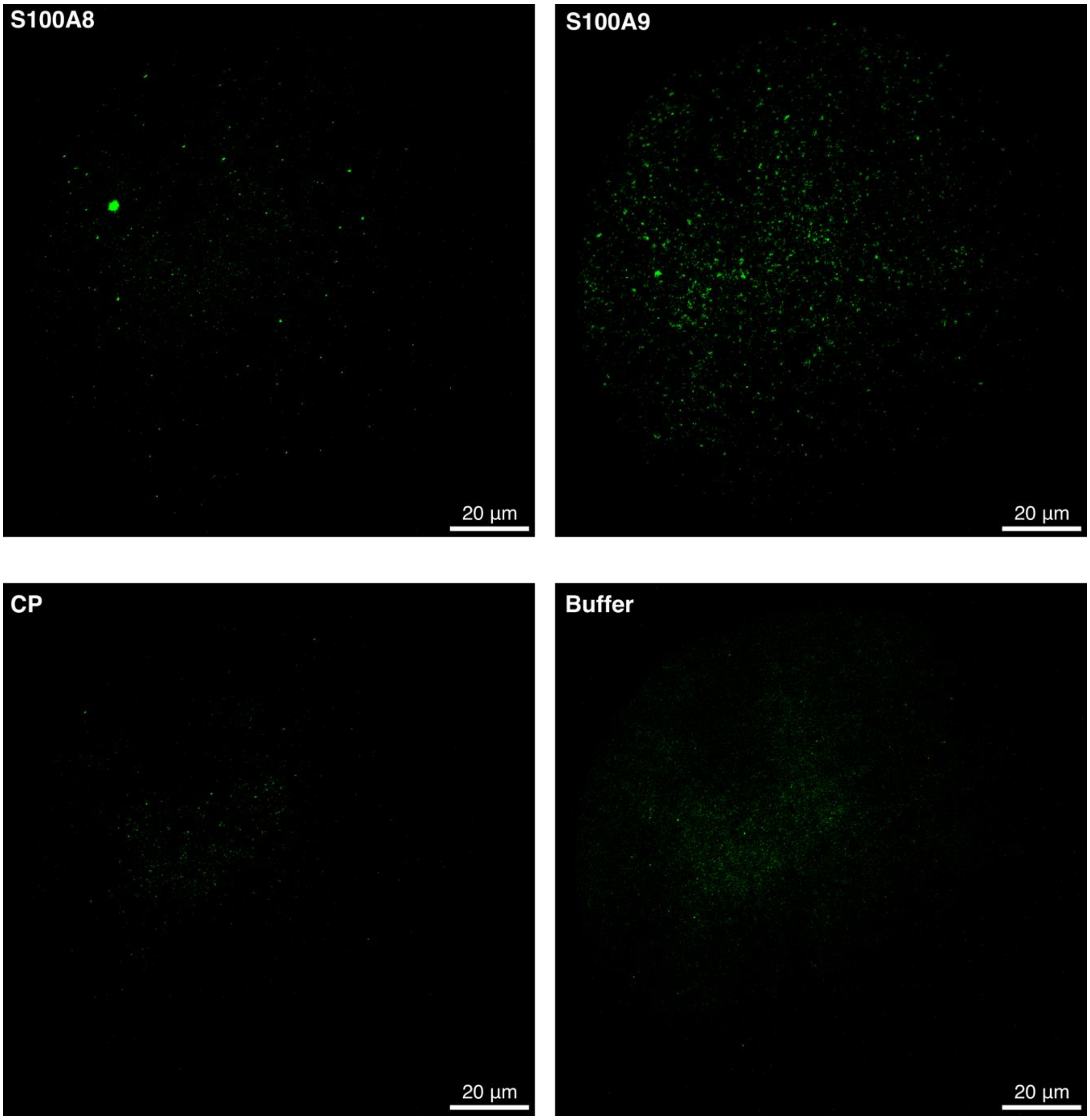

Figure S11. Uncropped fluorescence microscopy images of S100A8, S100A9, CP aggregates and buffer stained with ThS (scale bar, 20 μm).

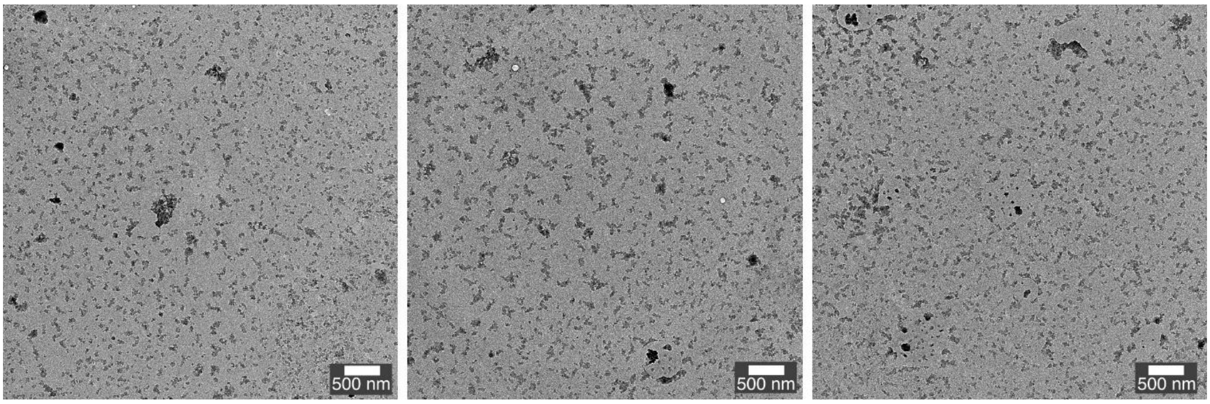

Figure S12. Transmission electron microscopy images of S100A8 aggregates stained with uranyl acetate (scale bar, 500 nm).

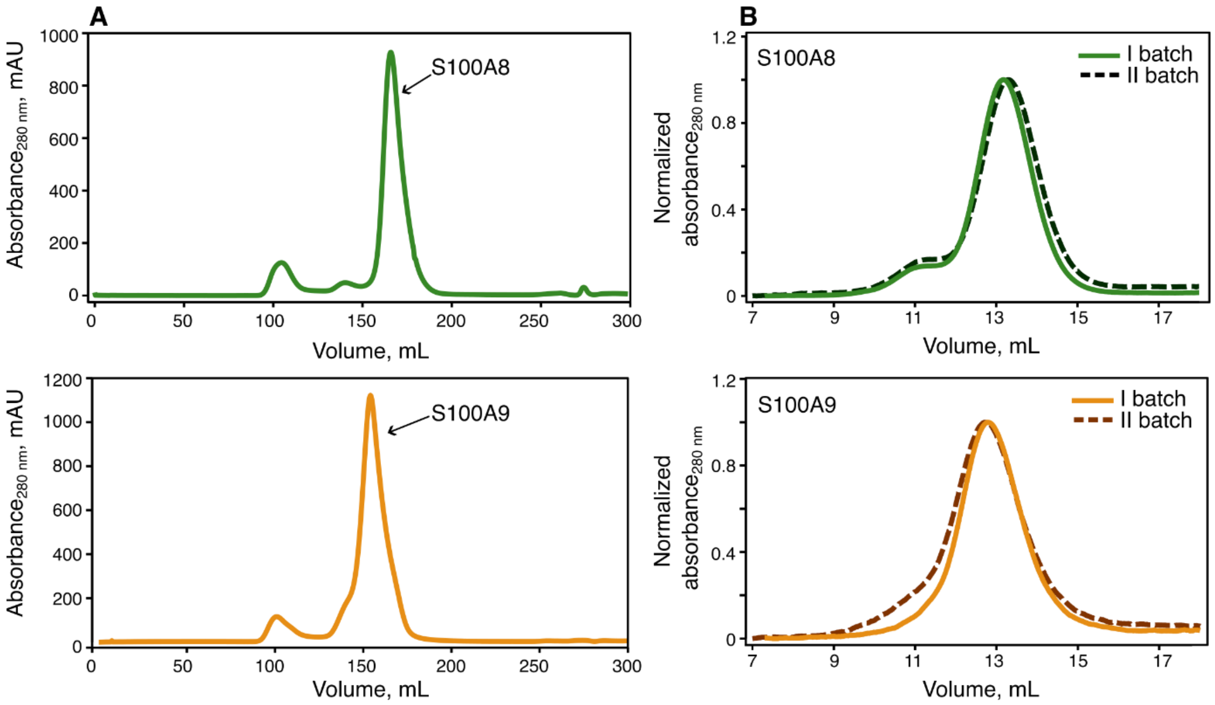

Figure S13. Size exclusion profiles of S100A8 and S100A9 during purification using HiLoad™ 26/600 column Superdex™ 75 prep grade column (Cytiva) (**A**). Comparison of size exclusion profiles between different S100A8 and S100A9 purification batches (analyzed on Tricorn™ 10/300 column (Cytiva), packed with Superdex™ 75 prep grade resin (Cytiva)) (**B**)

Table S1. Plasmids and respective primers used in this study.

Plasmid	Primer	Sequence
pDS98 (His_6-SUMO-S100A8)	pet15_5206	5' ATCGAGATCTCGATCCCGCG 3'
	DS98_S100A8_frw	5' GATTGGCGGTATGTTGACCGAGCTGGAGAA 3'
	DS98_S100A8_rev	5' CGGTCAACATACCGCCAATCTGTTCCAGATG 3'
	DS98_S100A8_BamHI	5 'CGGGATCCCACTACTCTTTGTGGCTTTCTT 3'
pDS99 (His_6-SUMO-S100A9)	pet15_5206	5' ATCGAGATCTCGATCCCGCG 3'
	DS99_S100A9_BamHI	5' CGGGATCCTTAGGGGGTGCCCTCCCC 3'
	DS99_S100A9_frw	5' GATTGGCGGTATGACTTGCAAAATGTCGCAG 3'
	DS99_S100A9_rev	5' GCAAGTCATACCGCCAATCTGTTCCAGATG 3'
